# CIDP, myasthenia gravis, and membranous glomerulonephritis – three autoimmune disorders in one patient: a case report

**DOI:** 10.1186/s12883-018-1120-6

**Published:** 2018-08-14

**Authors:** Saskia Bolz, Andreas Totzeck, Kerstin Amann, Mark Stettner, Christoph Kleinschnitz, Tim Hagenacker

**Affiliations:** 1 Department of Neurology, Essen University Hospital, Hufelandstrasse 55, 45147 Essen, Germany; 2 Department of Nephropathology, University of Erlangen-Nürnberg, Krankenhausstr. 8-10, 91054 Erlangen, Germany

**Keywords:** Autoimmunity, T cells, Autoantibodies, Neuromuscular, Neuroinflammation

## Abstract

**Background:**

We present a patient fulfilling the electrophysiological criteria for definite chronic inflammatory demyelinating polyneuropathy (CIDP), antibody-positive myasthenia gravis (MG), and membranous glomerulonephritis (MGN) confirmed by biopsy. To our knowledge, this is the first case of the concomitant appearance of these three autoimmune diseases in a single patient.

**Case representation:**

A 42-year-old Caucasian male presented with rapidly progressive gait disturbance, distal weakness of the lower extremities, ascending hypoesthesia, impaired fine motor skills, and beginning cranial nerve palsy showing dysarthrophonia, facial paralysis, and eye movement abnormalities and was diagnosed as rapid onset (atypical) CIDP. After 3 months, the patient complained of increasing physical exhaustion, reduction of his walking distance, worsening of the residual dysphagia, and dysarthria with an inability to swallow. AChR antibodies (17.0 nmol/L, RF < 0.4) and titin antibodies were positive and repetitive nerve stimulation showed an abnormal decrement matching the criteria of myasthenia gravis. Over time the patient developed severe acute-on-chronic renal failure with high-grade proteinuria resulting in generalized edema followed by secondary hyperparathyroidism and dialysis-dependent renal failure. Renal biopsy confirmed beginning anti-phospholipase A2 receptor antibody membranous nephropathy.

**Conclusion:**

All three diseases are of autoimmune origin with distinctive immunopathogenetic mechanisms. The present case of CIDP, MG, and MGN occurring in one patient indicates a common underlying immune mechanism in these distinct conditions, including the involvement of autoantibodies and T cells.

## Background

The concomitant occurrence of chronic inflammatory demyelinating polyneuropathy (CIDP) and myasthenia gravis (MG) has been sporadically reported [1–5]. Both diseases are of autoimmune origin with distinctive immunopathogenetic mechanisms. CIDP is an acquired demyelinating disease of the peripheral nervous system characterized by a slow conduction velocity and prolonged distal latencies in the motor and sensory nerve fibers. While it is well established that soluble factors such as complement and antibodies and macrophages and neuritogenic T cells act together to cause demyelination, the precise antigen and interaction between the aforementioned immune components remain largely undefined [6]. A characteristic feature of MG is fluctuating muscle weakness with an ocular onset or a generalized pattern. Autoantibodies against acetylcholine receptor (AChR) or low-density lipoprotein receptor-related protein 4 (LRP4) cause a T cell-dependent, complement-mediated, and mainly IgG-driven destruction of the postsynaptic muscular proteins as well as a competition with ACh on its receptors [7].

Thymic pathologies are considered to cause an aberrant autoimmune response in the few known cases of simultaneous MG and membranous glomerulonephritis (MGN). We present a patient fulfilling the electrophysiological criteria for definite CIDP, antibody-positive MG, and MGN confirmed by biopsy. To our knowledge, this is the first case of the concomitant appearance of the aforementioned three autoimmune diseases in a single patient and may indicate an underlying link among the different pathogeneses.

## Case presentation

**Fig. 1:**
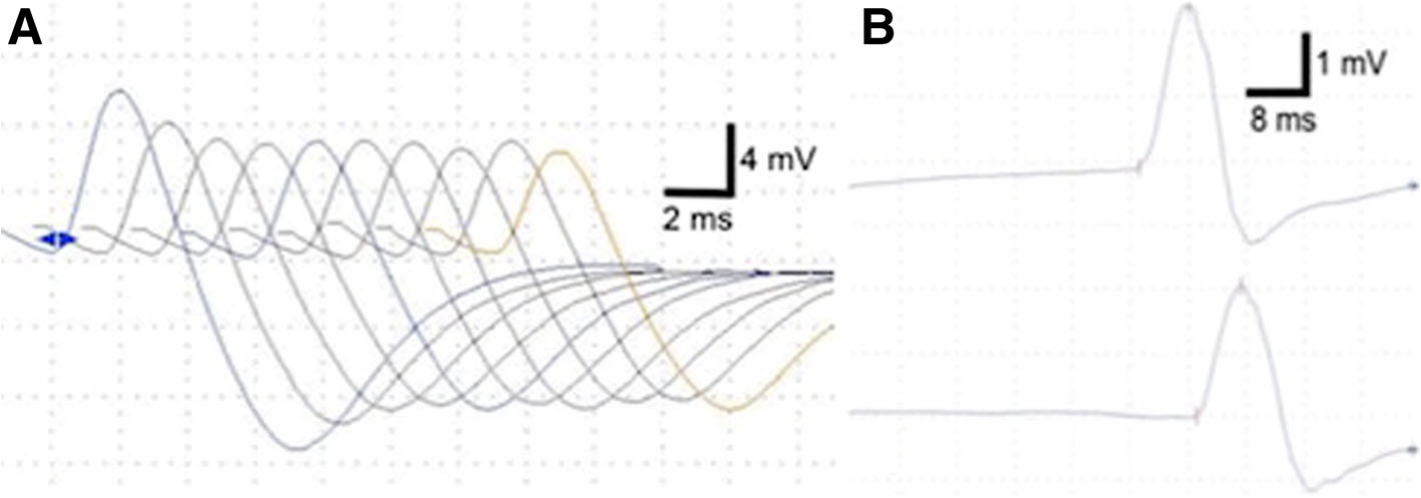
Repetitive nerve stimulation of the trapezius muscle (**a**) revealed a decrement as an electrophysiological sign of MG. The patient showed decreased motor nerve conduction velocities and prolonged distal motor latencies (of the median nerve as displayed here in (**b**)) as well as the absence of F-waves in the N. medianus and N. ulnaris, consistent with the diagnosis of a demyelinating polyneuropathy

A 42-year-old Caucasian male presented with rapidly progressive gait disturbance, distal weakness of the lower extremities, ascending hypoesthesia, impaired fine motor skills, and beginning cranial nerve palsy showing dysarthrophonia, facial paralysis, and eye movement abnormalities within several months. Tendon reflexes of the upper extremities were reduced and absent in the lower extremities. Nerve conduction studies showed a proximal demyelinating sensorimotor polyneuropathy with active denervation, prolonged motor distal latency, and a reduction in the motor conduction velocity in N. medianus and N. ulnaris (Fig. 1b). The cerebrospinal fluid (CSF) revealed albuminocytologic dissociation with an elevated protein level of 22,300 g/L (reference value [RF] < 0.4 g/L). Ganglioside antibodies (GD1a, GM1, GM2, GQ1b, and GT1b) were negative. Nerve conduction studies showed proximal demyelinating sensorimotor polyneuropathy with active denervation. With progressing symptoms showing no response to intravenous methylprednisolone, the patient was transferred to our intensive care unit (ICU). Treatment with intravenous immunoglobulin (IVIg, 30 g/day for 5 days) was initiated and the patient rapidly improved. Due to the clinical course of the disease and according to the EFNS/PNS criteria, the patient was diagnosed with typical CIDP.

Three months after discharge, the patient developed dysphagia, facial nerve palsy, dyspnea, and paralysis of the lower extremities. Under combined treatment with IVIg and methylprednisolone, the patient improved and was discharged to rehabilitation care. There, he complained about increasing physical exhaustion, reduction of his walking distance, worsening of the residual dysphagia, and dysarthria with an inability to swallow. His AChR antibodies (17.0 nmol/L, RF < 0.4) and titin antibodies were positive and repetitive nerve stimulation showed an abnormal decrement (Fig. 1a) matching the criteria of MG.

The patient’s symptoms improved after the administration of pyridostigmine. Computer tomography of the upper chest revealed an inhomogeneous mass in the anterior mediastinum. Biopsy confirmed a thymoma, which was subsequently managed by a thymectomy.

Follow-up visits displayed consistent albuminocytologic dissociation in the CSF and the AChR antibodies decreased (2.1 nmol/L). Under long-term immunosuppression with azathioprine 150 mg and prednisone 15 mg daily and another IVIg application, dysphagia, dysarthria, and gait unsteadiness consistently improved.

**Fig. 2:**
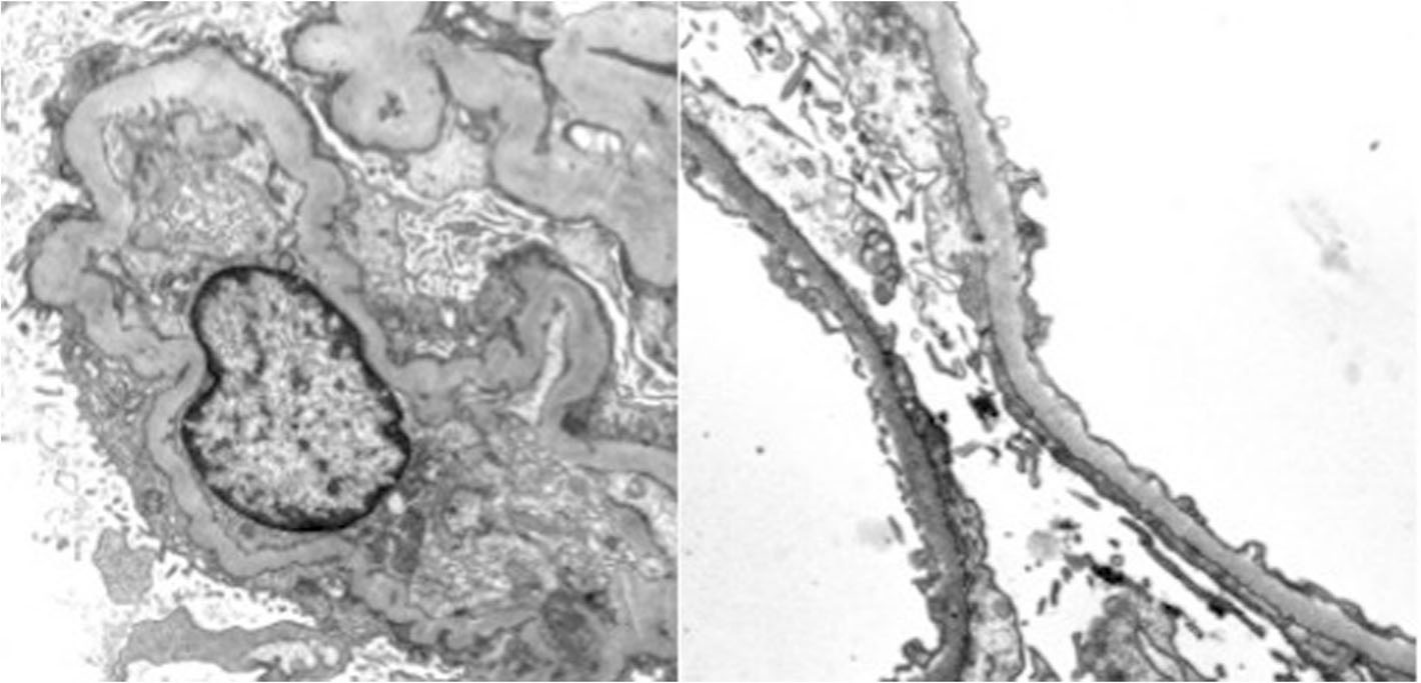
Electron microscopy of the renal biopsy confirmed the diagnosis of membranous glomerulonephritis with few subepithelial osmiophilic deposits (courtesy of Prof. Kerstin Amann, Dept. of Nephropathology, University of Erlangen-Nürnberg)

However, almost 2 years later, the patient developed severe acute-on-chronic renal failure with high-grade proteinuria resulting in generalized edema followed by secondary hyperparathyroidism and dialysis-dependent renal failure. Renal biopsy confirmed beginning anti-phospholipase A2 receptor antibody-positive membranous glomerulonephritis (MGN), a common cause of nephrotic syndrome (Fig. 2). One year of critical illness complicated by sepsis led to myasthenic crisis, but the patient recovered under repeated IVIg therapy and finally regained the partial ability to live autonomously.

## Discussion and conclusions

We present a case of three autoimmune disorders occurring in the same patient: CIDP, MG, and MGN.

Evidence from pathological studies leads to the consensus that T cell-mediated attack contributes to the pathogenesis of CIDP and an antigen-driven, major histocompatibility complex restricted T cell-mediated attack [8]. Autoantibodies against different antigens have been reported in a proportion of CIDP patients with high range variability. Humoral factors seem to play a role in CIDP because of the fast response to plasmapheresis, suggesting a circulating factor causing functional impairment [9].

An antigen-driven T cell response occurs in MG. Regulatory T cells (Tregs) and CD4+ T cells bind to AChR epitopes, stimulating B cells to produce antibodies [10].

The concomitant occurrence of CIDP and MG is rare. Isolated case reports suggest either an autoantibody similar to AChR autoantibody [11] or an imbalance of T helper cells causing these autoimmune disorders [2].

MGN is one main cause of nephrotic syndrome and typically shows subepithelial immune complex and complement deposits of IgG and C3c in histological biopsies. The deposition is followed by complement activation causing sublytic damage presenting as proteinuria, hypertension, or microscopic hematuria.

Interestingly, subtypes of CIDP, anti-phospholipase A2 receptor antibody-positive MGN, and MuSK MG are considered IgG4-mediated autoimmunopathies, whereas in our patient, MuSK antibodies were not detectable [12].

The occurrence of MGN in patients with MG has recently been reported [13] but is not considered a classical immunologic disorder that may accompany MG such as systemic lupus erythematosus. AChR antibodies may also bind to glomerular antigens leading to complement activation similar to their reaction in the neuromuscular junction [13]. Furthermore, thymus pathologies could cause renal disease, as it is associated with several autoimmune disorders, probably due to the induction of autoreactive T cells or the suppression of Tregs during the immune response [14]. Abnormal thymus tissue may trigger an autoimmune response resulting in an imbalance of immunoregulatory mechanisms [15]. However, several cases of glomerulonephritis without MG have been reported in patients with thymoma [13]. The present case of CIDP, MG, and MGN occurring in one patient indicates a common underlying immune mechanism in these distinct conditions, including the involvement of autoantibodies and T cells.

## Abbreviations

Ach: Acetylcholine
AChR: Acetylcholine receptor
CIDP: Chronic inflammatory demyelinating polyneuropathy
CSF: Cerebrospinal fluid
GBS: Guillain-Barré syndrome
ICU: Intensive care unit
IVIg: Intravenous immunoglobulin
LRP4: Low-density lipoprotein receptor-related protein 4
MG: Myasthenia gravis
MGN: Membranous glomerulonephritis
MuSK: Muscle-specific kinase
RF: Reference value
Tregs: Regulator T cells
